# Prognosis of schizophrenia in persons with and without a history of cannabis use

**DOI:** 10.1017/S0033291714000191

**Published:** 2014-02-19

**Authors:** E. Manrique-Garcia, S. Zammit, C. Dalman, T. Hemmingsson, S. Andreasson, P. Allebeck

**Affiliations:** 1 Department of Public Health Sciences, Division of Social Medicine, Karolinska Institutet, Stockholm, Sweden; 2 Department of Psychological Medicine and Neurology, MRC Centre for Neuropsychiatric Genetics and Genomics, Cardiff University, Cardiff, UK; 3 Department of Public Health Sciences, Division of Public Health Epidemiology, Karolinska Institutet, Stockholm, Sweden; 4 Institute of Environmental Medicine, Karolinska Institutet, Stockholm, Sweden; 5 Centre for Social Research on Alcohol and Drugs, Stockholm University, Stockholm, Sweden

**Keywords:** Cannabis, prognosis, psychosis, schizophrenia

## Abstract

**Background:**

The aim of the study was to determinate whether schizophrenia patients with a history of cannabis use have a different prognosis, with regards to readmission and hospital duration, compared with those without a history of cannabis use.

**Method:**

The present investigation was a cohort study of 50 087 Swedish men with data on cannabis use at the ages of 18–20 years. A total of 357 cases of schizophrenia were identified from in-patient care and followed up from 1973 to 2007.

**Results:**

Schizophrenia patients with a history of cannabis use had a higher median duration of first hospital episode (59 days *v.* 30 days). Patients with a history of cannabis use had a higher median rate of readmission (10 times *v.* four times). Also, total number of hospital days was higher in patients with a history of cannabis use compared with those without (547 days *v.* 184 days). Patients with a history of cannabis use had an increased odds of having more than 20 hospital readmissions compared with non-users [3.1, 95% confidence interval (CI) 1.3–7.3] as well as an increased odds of hospital admission lasting more than 2 years (2.4, 95% CI 1.1–7.4) after controlling for diagnosis of personality disorders, family socio-economic position, IQ score, civil status, place of residence, risky use of alcohol and use of other drugs. Patients with a history of cannabis use were less likely to have paranoid schizophrenia compared with never users (8% *v.* 17%) in the first admission.

**Conclusions:**

Schizophrenia patients with a history of cannabis use had a significantly higher burden of lifetime in-patient care than non-cannabis users. Not only does cannabis increase the risk of schizophrenia, but also our findings indicate that the course and prognosis of schizophrenia may be more severe than schizophrenia cases in general.

## Introduction

While evidence has grown stronger in recent years that use of cannabis in adolescence is associated with later incidence of schizophrenia (Moore *et al.* 2007; Hall & Degenhardt, 2009), several issues need to be clarified regarding this association. It would be of clinical as well as public health importance to find out whether schizophrenia associated with cannabis use has specific prodromal characteristics, if these schizophrenia cases have specific clinical characteristics and if the course of illness differs from schizophrenia not associated with cannabis use.

In most cases, schizophrenia is preceded by a prodromal phase that can be manifested as non-specific clinical states, with depressive and anxiety symptoms (Buckley *et al.* 2009), which can last up to several years (an der Heiden & Hafner, 2000; Rosen *et al.* 2006). These prodromal symptoms are non-specific of schizophrenia, and most people with these symptoms will not develop schizophrenia. The prodromal phase of schizophrenia can also include psychotic features, and it is indeed common that many persons, who later receive a diagnosis of schizophrenia, previously have been given a diagnosis of brief psychosis or other non-affective psychosis (Castagnini & Berrios, 2009).

While it is widely agreed that the entity of schizophrenia in itself as well as its various subtypes are heterogeneous and not easily classified (van Os & Kapur, 2009), methods such as latent class analysis (Castle *et al.* 1994) and grade of membership analysis have indicated that a distinction between at least a paranoid type and a hebephrenic type can be made, and sometimes also a simple type (Pomarol-Clotet *et al.* 2010). Thus, when trying to identify subtypes of schizophrenia that might be particularly associated with cannabis use, standard International Classification of Diseases (ICD) classification used in clinical practice may still be valuable. In a previous study (Andreasson *et al.* 1989), we found that those patients with schizophrenia who had a history of cannabis use had a more abrupt onset and more positive symptoms than those without. It would be interesting to find out whether such a difference in symptoms would be reflected in different diagnostic subtypes.

Several studies have addressed the effect of cannabis on the course of illness in patients with schizophrenia. Zammit *et al.* (2008) reviewed longitudinal studies of people with psychosis and found that cannabis use was consistently associated with increased relapse or rehospitalization, and poorer adherence to treatment. They noted, however, that many studies had limitations, such as lack of control for baseline severity, and confounding. In order to overcome methodological limitations, Foti *et al.* (2010) followed 229 patients with schizophrenia for 10 years with the aim of examining the association between cannabis use and course of illness. They concluded that cannabis is associated with an adverse course of psychotic symptoms in schizophrenia, but that the association was bidirectional, i.e. psychotic symptoms also increased cannabis use. Thus, while there is substantial evidence on the role of cannabis in persons with established psychoses, it would be of clinical importance to know whether the course and outcome of schizophrenia differ in people with a history of cannabis use prior to the onset of schizophrenia compared with those without.

The Swedish conscript survey is, to date, the longest follow-up of psychotic patients with data on cannabis use prior to incidence of psychosis. The conscript cohort has been used previously to examine the association between cannabis and schizophrenia (Andreasson *et al.* 1987; Zammit *et al.* 2002), and showed support for a causal effect of cannabis leading to an increased risk of developing schizophrenia. This finding has been supported by other longitudinal studies such those in the Netherlands (van Os *et al.* 2002), Germany (Henquet *et al.* 2005) and New Zealand (Arseneault *et al.* 2002; Fergusson *et al.* 2003), as summarized in a systematic review by Moore *et al.* (2007). The aim of the present study, based on all 401 persons with schizophrenia in this Swedish cohort, was to assess the characteristics and course of disease among patients with a history of cannabis use compared with those without. Our specific aims were to find out:

(1) Whether, in a sample of individuals with schizophrenia, there is any difference in pre-morbid psychiatric diagnosis among patients with a history of cannabis use compared with those without a history of cannabis use;
(2) Whether there is any difference in types of schizophrenia among patients with a history of cannabis use compared with non-users;
(3) Whether there is any difference in the duration of first admission for schizophrenia among patients with a history of cannabis use compared with non-users;
(4) Whether schizophrenia patients with a history of cannabis use have a different prognosis, with regard to readmissions and hospital duration, compared with those without a history of cannabis use.

## Method

The cohort consisted of 50 087 Swedish men who were conscripted during 1 year (1969–1970) for compulsory military training. Over 93% of the men were aged 18–19 years. Only 2–3% of men were exempted from conscription mainly because of a severe handicap or a congenital disorder. All of the men completed two self-report non-anonymous questionnaires at the time of conscription. The first questionnaire concerned social upbringing conditions, friendship, relationships, attitudes, and adjustment at school and work. The second questionnaire concerned the use of alcohol, tobacco, drugs and substance use.

All conscripts were assessed by a psychologist after a structured interview and psychological test as well as an intelligence quotient (IQ) test. Those presenting with psychiatric symptoms were referred to a psychiatrist and any psychiatric disorders found were diagnosed according to the ICD, eight revision (ICD-8). Permission to use the conscription database for research purposes and to perform the relevant record linkages was granted by the Stockholm Regional Ethical Review Board.

### Exposure

Information on cannabis use was obtained from the conscription survey at the time of conscription. Questions were asked whether subjects had ever used drugs, which drugs had ever been used, first drug used, drug most commonly used, frequency of use, and questions regarding use of specific drugs from a list with alternatives.

Cannabis use was categorized as a dichotomous variable ever used *versus* never used cannabis.

### Follow-up

The cohort was followed in the Swedish National In-patient Register, which records all in-patient admissions to hospitals in Sweden from 1973 until 2007. Diagnoses were coded according to the Swedish version of the eighth revision (ICD-8; 1965–1986), ninth revision (ICD-9; 1987–1996) and tenth revision (ICD-10; 1997–2007). The following diagnostic codes were used for schizophrenia: 295 according to ICD-8 (excluding 295.50, 295.70), 295 according to ICD-9 (excluding 295F, 295H) and F20 according to ICD-10.

Data were linked to Sweden's National Cause of Death Register and the Swedish Migration Register. We followed up participants from 1 January 1973 until 31 December 2007. The date of first emigration and day of death were used as censoring points. Apart from subjects who emigrated (four persons) or died (82 persons), no participants were lost to follow-up.

### Outcomes

#### First pre-morbid psychiatric diagnosis

As an indicator of pre-morbid characteristics, we assessed diagnoses received by patients prior to first diagnosis of schizophrenia, classified as follows:

(*a*) Brief psychosis: 294.30 psychosis associated with other physical conditions/drug or poison intoxication (ICD-8); 298 reactive psychosis (ICD-8 and ICD-9); 292 drug psychosis (ICD-9); 293 transient organic psychotic conditions (ICD-9); F125 and F127 psychotic disorder due to use of cannabinoids (ICD-10); F23 acute and transient psychotic disorders (ICD-10).
(*b*) Schizo-affective disorder: 295.70 schizophrenia type schizo-affective (ICD-8); 295H schizo-affective form (ICD-9); and F25 schizo-affective disorders (ICD 10).
(*c*) Other psychoses: 299.99 unspecified psychoses (ICD-8); 297 paranoia states (ICD-8 and ICD-9); F28 other non-organic psychotic disorders (ICD-10); F29 unspecified non-organic psychosis (ICD-10); F22 persistent delusional disorders (ICD-10).
(*d*) Neurosis: 300.00 to 300.99 (ICD-8); 300A to 300X (ICD-9); F400 to F489 (ICD-10).
(*e*) Personality disorders: 301.00 to 301.99 (ICD-8); 301A to 301X (ICD-9); and F600 to F690 (ICD-10).

#### Subtype of schizophrenia at first admission

We assessed type of schizophrenia diagnosed at first admission as follows:

(*a*) Simple schizophrenia: 295.00 (ICD-8); 295A (ICD-9); F206 (ICE-10);
(*b*) Hebephrenic schizophrenia: 295.10 (ICD-8); 295B (ICD-9); F201 (ICE-10);
(*c*) Catatonic schizophrenia: 295.20 (ICD-8); 295C (ICD-9); F202 (ICE-10);
(*d*) Paranoid schizophrenia: 295.30 (ICD-8); 295D (ICD-9); F200 (ICE-10);
(*e*) Acute schizophrenia: 295.40 (ICD-8); 295E (ICD-9);
(*f*) Other schizophrenia: 295.80, 295.99 (ICD-8); 295W, 295X (ICD-9); F208, F209, F203, F204 (ICD-10).

#### Duration of first admission

From data in the in-patient register, we assessed the median number of hospital days recorded for the first admission with a diagnosis of schizophrenia.

#### Total number of hospital days

We assessed the median total number of hospital days in psychiatric care from the first admission to last discharge or end of follow-up. In the regression analyses taking into account confounders, we categorized the duration in four groups: <45 days; 46–179 days; 180–729 days; and >730 days.

#### Number of readmissions

We assessed the median number of readmissions. In the regression analyses, we categorized them in three groups: <4 times; 5 to 20 times; >20 times.

### Sensitivity analysis

Since substance use disorder often led to complications necessitating psychiatric care, we performed sensitivity analyses excluding subjects with diagnoses of other substance use disorder during the follow-up. Diagnoses of substance use disorder were obtained from the in-patient register (1973 to 2010). The following diagnostic codes were used: drug dependence (304 according to ICD-8 and ICD-9), and dependence syndrome of opioids (F112), sedatives or hypnotics (F132), cocaine (F142), other stimulants (F152), hallucinogens (F162), volatile solvents (F182), and other psychoactive substances (F192) according to ICD-10.

Considering that cannabis use has been associated with an early age at onset of schizophrenia, we also performed analyses on hospital admissions and number of days adjusting for age at first admission for schizophrenia.

### Covariates

We selected potential confounding variables on the basis of prior research indicating that they are likely to be associated with both cannabis use and the prognosis of schizophrenia, with regard to total number of hospital days and number of readmissions.

#### Diagnosis of personality disorders at baseline

Diagnosis of personality disorders was assessed by a psychiatrist at conscription: any *versus* none.

#### Family socio-economic position

Information on family socio-economic position was based on Census 1960 on data on each conscript's father's occupation: non-manual (collapsed intermediate and high non-manual), low non-manual, manual (unskilled, skilled) and others (farmers, self-employed, unclassified).

#### IQ score at conscription

IQ score consisted of four main subtest parts (verbal IQ, visuospatial ability, general knowledge and mechanical ability); these four subtests were aggregated to give an overall standardized intelligence score, which we collapsed into a composite standard three-category scale: highest (111 to >126) *versus* middle (90 to 110) and lowest (<74 to 89).

#### Civil status during the follow-up

Information on civil status was based on Census 1970, 1975, 1980, 1985 and 1990. We categorized as a dichotomous variable ever married *versus* never married.

#### Place of residence at conscription

Information on place of residence was obtained at time of conscription: any one of Sweden's three large metropolitan areas (Stockholm, Gothenburg, Malmö) *versus* the rest of the areas.

#### Risky use of alcohol at conscription

Information on risky use of alcohol was derived from questions on high consumption of alcohol: none versus at least one of the following indicators – consumption of at least 250 g 100% alcohol/week; have taken an ‘eye-opener’ during a hangover; have been apprehended for drunkenness; have reported being drunk often.

#### Use of other drugs at conscription

Information on use of other drugs was obtained at the time of conscription, including amphetamine, lysergic acid diethylamide, morphine, mebumal and opium: any versus none.

### Data analysis

In the analysis of first pre-morbid psychiatric diagnosis, we excluded those subjects who received a diagnosis of psychiatric disorder at conscription, in order to minimize possibility of reverse causality. In the analyses of duration of first admission, total number of hospital days and number of hospital readmissions, we excluded those subjects who were admitted to a forensic regional healthcare facility for criminal conviction during the follow-up, since the hospital stay was determined by the judicial system.

Differences in first pre-morbid psychiatric diagnosis of schizophrenia and type of schizophrenia at first admission were tested using Fisher's exact test. The Wilcoxon signed test was used to test the difference in the medians for longer duration of first admission, high total number of hospital days and high number of readmission among subjects who had ever used cannabis compared with those who had never used.

Negative binomial regression was used to estimate rate ratios with 95% confidence intervals (95% CIs) for duration of first admission, total number of hospital days and number of readmission during the 34 years of follow-up among subjects who had ever used cannabis compared with those who had never used. This method is for modelling count variables instead of dichotomous outcomes. In order to control for specifying length of follow-up time, we adjusted for age at first admission for schizophrenia. Negative binomial regression was used instead of Poisson regression because of over-dispersion of the variance relative to the mean during the follow-up (Lachin, 2011).

In order to control for other factors that influence prognosis, we adjusted for the potential confounding factors mentioned above. Here, multinomial logistic regression was performed using defined cut-off points as outcomes. Odds ratios (ORs) were computed with 95% CIs. We report both crude results and results adjusted for confounders. The analyses were performed in SAS 9.1 for Windows (SAS Institute Inc., USA).

## Results

In the whole cohort of 50 087 conscripts, we identified 401 (0.8%) subjects with a diagnosis of schizophrenia during the follow-up period. We excluded 44 individuals who did not respond to the question on drug use at conscription. Among the remaining 357 subjects, 72 (20%) individuals reported having a history of cannabis use. There were no significant differences in the mean follow-up period between patients with a history of cannabis use and non-users (21 years).

### First pre-morbid psychiatric diagnosis

Table 1 shows that a total of 144 (40%) out of the 357 patients with schizophrenia had preceding in-patient episodes with other psychiatric diagnoses. However, there was no evidence that pre-morbid psychiatric diagnosis differed between patients with a history of cannabis use and non-users.

**Table 1.** Pre-morbid psychiatric diagnoses of schizophrenia

	Total cases, *n*	Never used cannabis, *n* (%)	Ever used cannabis, *n* (%)	*p*
Brief psychosis	30	27 (22)	3 (14)	0.2
Schizo-affective disorders	4	3 (2)	1 (5)	0.2
Other psychoses	52	44 (36)	8 (36)	0.2
Neurosis	37	31 (25)	6 (27)	0.3
Personality disorders	21	17 (14)	4 (18)	0.2

### Subtype of schizophrenia at first admission

As shown in Table 2, schizophrenia patients without a history of cannabis use were more likely to have paranoid schizophrenia than ever users (17% *v.* 8%). There were no significant differences between patients with a history of cannabis use and non-users when comparing the other subtypes of schizophrenia.

**Table 2.** Subtype of schizophrenia at first admission

	Total, *n*	Never used cannabis, *n* (%)	Ever used cannabis, *n* (%)	*p*
Simple	37	29 (10)	8 (11)	0.2
Hebephrenic	22	16 (6)	6 (8)	0.1
Catatonic	7	5 (2)	2 (3)	0.3
Paranoid	56	50 (17)	6 (8)	0.02
Acute	6	4 (2)	2 (3)	0.2
Other	229	181 (63)	48 (67)	0.09

### Duration of first admission, total number of hospital days, and number of readmissions

Table 3 shows that schizophrenia patients with a history of cannabis use had a higher median duration of first hospital episode (59 days *v.* 30 days) and higher median number of readmissions (10 times *v.* four times). Also the total number of hospital days was higher in patients with a history of cannabis use compared with those without (547 days *v.* 184 days). The associations persisted after excluding those with diagnoses of substance use disorder during the follow-up.

**Table 3.** Median duration of first hospital episode, number of readmissions and total number of hospital days among subjects reporting ever use versus never use of cannabis

	All subjects			Excluding subjects with diagnoses of other substance use disorder		
	Never used cannabis	Ever used cannabis	*p*	Never used cannabis	Ever used cannabis	*p*
Median duration of first hospital episode, days	30	59	0.01	28	66	0.01
Median number of readmissions	4	10	<0.01	4	7	0.04
Median total number of hospital days	184	547	<0.01	157	266	0.04

Table 4 shows that patients with a history of cannabis use had an increased rate ratio of longer duration of first admission (1.4, 95% CI 1.3–1.5), a higher total number of hospital days (1.5, 95% CI 1.4–1.6) and a higher number of readmissions (1.5, 95% CI 1.4–1.6) compared with non-users during the whole follow-up. The associations persisted after adjusting for age at first admission [duration of first admission (1.4, 95% CI 1.1–2.8), total number of hospital days (1.5, 95% CI 1.1–2.4) and number of readmissions (1.3, 95% CI 1.1–2.8)].

**Table 4.** Rate ratios for duration of first admission, total number of hospital days and number of readmissions during the 34 years of follow-up^a^

	Events, *n*	Person-years	Crude rate ratio (95% CI)	Adjusted rate ratio (95% CI)^b^
Duration of first hospital episode				
Never used cannabis	25 305	5812	1	1
Ever used cannabis	9296	1460	1.4 (1.3–1.5)	1.4 (1.1–2.8)
Number of readmissions				
Never used cannabis	2332	3665	1	1
Ever used cannabis	955	1005	1.5 (1.3–1.6)	1.3 (1.1–1.8)
Total number of hospital days				
Never used cannabis	154 300	5382	1	1
Ever used cannabis	52 426	1207	1.5 (1.4–1.6)	1.5 (1.1–2.4)

CI, Confidence interval.

a Rate ratios were calculated by using negative binomial regression.

b Adjusting for age at first admission for schizophrenia.

Table 5 shows the distribution of different numbers of hospital days controlling for confounding factors. There was an almost 3-fold increased odds of hospital admission lasting more than 2 years (>730 days) in people who had used cannabis compared with those without cannabis use. This was only slightly attenuated after controlling for confounding (adjusted OR = 2.4, 95% CI 1.1–7.4).

**Table 5.** Odds ratios for total number of hospital days among schizophrenia patients^a^

	Hospital days			
	<45 days	46–179 days	180–729 days	>730 days
Never used cannabis, *n* (%)	71 (27)	63 (24)	71 (27)	56 (21)
Ever used cannabis, *n* (%)	11 (19)	12 (21)	13 (22)	22 (38)
Crude odds ratio (95% CI)	1 (ref)	1.2 (0.5–2.9)	1.3 (0.5–2.8)	2.5 (1.2–5.6)
Adjusted odds ratio (95% CI)^b^	1 (ref)	1.6 (0.6–4.2)	1.3 (0.5–3.7)	2.4 (1.1–7.4)

CI, Confidence interval; ref, reference.

a Odds ratios were calculated by using multinomial logistic regression.

b Adjusted for diagnosis of personality disorders at baseline, family socio-economic position, intelligent quotient score at conscription, civil status during the follow-up, place of residence at conscription, risky use of alcohol at conscription, and use of other drugs at conscription.

Table 6 shows the distribution of different numbers of readmissions controlling for confounding factors. There was an almost 4-fold increase in oddsof having more than 20 hospital readmissions in patients with a history of cannabis use compared with non-users. This was slightly reduced after adjustment for confounding (adjusted OR 3.1, 95% CI 1.3–7.3).

**Table 6.** Odds ratios for number of readmissions among schizophrenia patients^a^

	Readmissions		
	<4 times	5–20 times	>20 times
Never used cannabis, *n* (%)	138 (53)	96 (37)	27 (10)
Ever used cannabis, *n* (%)	25 (43)	16 (28)	17 (29)
Crude odds ratio (95% CI)	1 (ref)	1.1 (0.6–2.0)	3.4 (1.6–7.1)
Adjusted odds ratio (95% CI)^b^	1 (ref)	0.8 (0.4–1.7)	3.1 (1.3–7.3)

CI, Confidence interval; ref, reference.

a Odds ratios were calculated by using multinomial logistic regression.

b Adjusted for diagnosis of personality disorders at baseline, family socio-economic position, intelligent quotient score at conscription, civil status during the follow-up, place of residence at conscription, risky use of alcohol at conscription, and use of other drugs at conscription.

## Discussion

Results of this long-term (34 years) follow-up show that schizophrenia patients with a history of cannabis use had a significantly higher burden of in-patient care, with regard to hospital readmission and hospital duration, compared with those without a history of cannabis use. The association persisted after controlling for confounders, with a two-fold increased risk of having more than 2 years of hospital admissions, and a three-fold increased risk of having more than 20 hospital readmissions in patients with a history of cannabis use compared with non-users. Even the duration of first admission among subjects with a history of cannabis use was longer than for non-users, indicating early differences in prognosis between patients with a history of cannabis use and non-users.

Our findings may seem surprising, since a previous study from this cohort byAndreasson *et al.* (1989) indicated that patients with a history of cannabis use had a more abrupt onset with more positive symptoms compared with those without. Also in another longitudinal study of patients with cannabis dependence in Stockholm County (Allebeck *et al.* 1993), we found that schizophrenia patients with a history of cannabis use had a predominance of positive symptoms. In general, this would indicate a better prognosis (Remschmidt & Theisen, 2012), but the high number of readmissions and longer duration of hospital stay indicate poorer prognosis in these cases. We have not yet been able to analyse symptoms in more detail, but we plan to scrutinize medical records to assess clinical characteristics such as positive and negative symptoms in the two groups.

There are a number of possible mechanisms that can explain the associations between cannabis use and increased relapses and poorer clinical outcome in schizophrenia. It has been suggested that cannabis use can cause long-lasting dysregulation of the endogenous anandamide/cannabinoid system that mediates the effect of tetrahydrocannabinol within the brain (van Os *et al.* 2002). It has also been suggested that cannabis increases the number of cannabinoid receptors in the brain, causing an increased vulnerability for repeated psychotic episodes (Ferdinand *et al.* 2005). Cannabis use has also been found to correlate with poor compliance with medication in first-episode schizophrenia (Coldham *et al.* 2002; Kamali *et al.* 2006; Miller *et al.* 2009), which may result in poorer outcome.

We did not find evidence that schizophrenia patients with a history of cannabis use have a different pattern of pre-morbid psychiatric diagnosis prior to their first admission with schizophrenia, compared with those without a history of cannabis use. Thus, early psychiatric disorders might be just as common in patients with a history of cannabis use as in non-users, as previously found in this cohort (Lewis *et al.* 2000). Arendt *et al.* (2005) found a high incidence of later schizophrenia in patients with ‘cannabis psychosis’. While this diagnosis was not used in the older versions of the ICD, one might have expected a higher proportion of brief psychotic episodes among those with a history of cannabis use.

We found a lower proportion of paranoid schizophrenia among ever users compared with never users. Though the number of cases was small, a lower proportion of paranoid schizophrenia could be consistent with poorer prognosis since paranoid schizophrenia often is associated with better prognosis (Zalewski *et al.* 1998).

### Limitations

There are a number of limitations with our study, which means that results should be interpreted with some caution. First, the causal pathway between cannabis and schizophrenia is complicated, and even though cannabis use was assessed at baseline, we do not know to what extent subjects continued cannabis use into adulthood. It is possible that longer hospitalization and more readmissions were due to continued cannabis use among patients with schizophrenia. If this was the case, it would confirm previous findings by Zammit *et al.* (2008) of a poorer outcome of schizophrenia among cannabis users. Also first-time use of cannabis after the age at conscription is possible, although it seems unlikely that this would affect our conclusions since subjects who use cannabis during adolescence appear to have a worse prognosis of schizophrenia than subjects who use cannabis during adulthood (Marshall & Rathbone, 2011). Second, only men born between 1949 and 1951 were included in this study, which might limit the generalizability of our findings. However, while this cohort may not be representative of the total Swedish population or later cohorts, a homogeneous study population does enable analyses of lifetime prognosis of schizophrenia in relation to background factors. Replication in other populations, at other times, would be valuable, although such cohorts are rare, as shown by Moore *et al.* (2007). Third, we are limited in that we do not have data regarding treatment for schizophrenia in the in-patient register, cannabis is associated with a decreased adherence to treatment and further studies are needed in order to clarify if adherence to treatment explains the associations between cannabis use and poorer clinical outcome in schizophrenia, as suggested by Miller *et al.* (2009).

The validity of the data on cannabis use in the conscript surveys has been previously assessed and found to be adequate (Otto, 1980; Benson & Holmberg, 1985). Likewise, the diagnosis of schizophrenia in the in-patient register has been found to be valid (Dalman *et al.* 2002; Ekholm *et al.* 2005).

## Conclusions

In spite of its limitations, this study is the longest longitudinal study of schizophrenia patients with data on cannabis use at baseline and later incidence of schizophrenia. In addition to our previous findings of an increased risk of schizophrenia in subjects with history of cannabis use, we now show that schizophrenia patients with a history of cannabis use also have a poorer prognosis, as indicated by longer hospital episodes and more readmissions. Thus, it is of public health as well as clinical importance that, as well as increasing risk of schizophrenia, cannabis may also lead to an illness that is more severe than in non-users of this drug.
